# The dimensions of evolutionary potential in biological conservation

**DOI:** 10.1111/eva.12995

**Published:** 2020-06-12

**Authors:** Emmanuel Milot, Arnaud Béchet, Virginie Maris

**Affiliations:** ^1^ Department of Chemistry, Biochemistry and Physics Université du Québec à Trois‐Rivières Trois‐Rivières Québec Canada; ^2^ Tour du Valat Research Institute for the Conservation of Mediterranean Wetlands Arles France; ^3^ Centre d'écologie fonctionnelle et évolutive, CNRS, EPHE, IRD Univ Montpellier Univ Paul Valéry Montpellier 3 Montpellier France

**Keywords:** evolutionary conservation, fixism, genetic diversity, value of biodiversity

## Abstract

It is now well admitted by ecologists that the conservation of biodiversity should imply preserving the evolutionary processes that will permit its adaptation to ongoing and future environmental changes. This is attested by the ever‐growing reference to the *conservation of evolutionary potential* in the scientific literature. The impression that one may have when reading papers is that conserving evolutionary potential can only be a good thing, whatever biological system is under scrutiny. However, different objectives, such as maintaining species richness versus ecosystem services, may express different, when not conflicting, underlying values attributed to biodiversity. For instance, biodiversity can be intrinsically valued, as worth it to be conserved *per se*, or it can be conserved as a means for human flourishing. Consequently, both the concept of evolutionary potential and the prescriptions derived from the commitment to conserve it remain problematic, due to a lack of explicit mention of the norms underlying different conservation visions. Here, we contend that those who advocate for the conservation of evolutionary potential should position their conception along four dimensions: what vehicles instantiate the evolutionary potential relevant to their normative commitment; what temporality is involved; how measurable evolutionary potential is, and what degree of human influence is tolerated. We need to address these dimensions if we are to determine why and when the maintenance of evolutionary potential is an appropriate target for the conservation of biodiversity.

## 1 INTRODUCTION

“We can either chose to manage evolutionary processes or not, but evolutionary change will proceed regardless” (Smith et al., 2014)

Conservation biology has been criticized for adopting a fixist conception of life. For the sharpest critics (e.g. Ibisch & Jennings, 2005; Smith, Bruford, & Wayne, 1996), conservationists are freezing the evolution of life when they try to preserve existing species or communities just as they are. However, the truth is conservation scientists have been strongly infused by evolutionism. In his seminal article published in 1985, Michael Soulé wrote that conservationists care for “the long‐range viability of whole systems and species, including their evolutionary potential” (Soulé, 1985). Subsequent contributions have awakened ecologists to the idea that conserving natural biological systems over the long term implies preserving their capacity to cope with spatio‐temporal variation of their environment, namely to *evolve* and *adapt* (e.g. Carroll & Fox, 2008; Eizaguirre & Baltazar‐Soares, 2014; Ferrière, Dieckmann, & Couvet, 2004; Frankham, Ballou, & Briscoe, 2002; Ryder, 1986). Basic evolutionary biology teaches us that the genetic composition of populations is constantly changing by the means of mutation, drift, migration and natural selection. Therefore, conservation actions that fail to take these forces into account may compromise fundamental processes creating and sustaining biodiversity (Hendry et al., 2010; Ibisch & Jennings, 2005; Smith, Bruford, & Wayne, 1996). Calls for the conservation of evolutionary potential (CEP) have multiplied in front of the growing impact of human activities on the planet (Harrisson, Pavlova, Telonis‐Scott, & Sunnucks, 2014; Hoelzel, Bruford, & Fleischer, 2019; Robert et al., 2017; Sarrazin & Lecomte, 2016). Unfortunately, CEP has also become a sort of catchall concept, and it is not always obvious how it contributes to biodiversity conservation practice (Box 1).

At least two different **epistemic intuitions** (see Glossary in Box 2) underlie the motivation to conserve **evolutionary potential** *:* (a) Evolution is the diversifying process that has shaped the actual biodiversity, from genes to ecosystems; thus, protecting the diversifying process is a way to maintain the capacity of biodiversity to evolve. We call this vision the *biodiversity‐generating process* perspective; (b) evolution is the adaptation process by which biological entities may respond to new selective pressures—such as climate changes—so conserving the adaptation process is a way to allow biological entities to persist through time. We call this vision the *biodiversity‐pattern* perspective. These two epistemic intuitions are intertwined with a variety of **normative intuitions** regarding the ultimate reasons why biodiversity should be conserved. Indeed, conservation ethics appeals to a broad spectrum of **normative values**, from the intrinsic value of nature to the anthropocentric values of ecosystem services (Box 3).

Both the biodiversity‐generating process perspective and the biodiversity‐pattern perspective appear convergent at the scale of global biodiversity. However, the *concept* of evolutionary potential (EP) and the *prescriptions* derived from the commitment to conserve it remain ill‐defined, sowing confusion and possibly participating in the limited adoption of evolutionary principles in conservation policies (Mace & Purvis, 2008). Thus, a first challenge is to formulate an appropriate definition of EP. In that matter, several similar expressions are used in the literature: evolutionary potential, evolvability, adaptive potential, adaptability (e.g. Conrad, 2012; Masel & Trotter, 2010; Mittell, Nakagawa, & Hadfield, 2015). Each of these expressions can take various meanings, sometimes vague, sometimes precise, depending on the studied object. For example, evolutionary potential can refer to mutation rate (EP of the genome), genetic variance (EP of traits and populations), species diversity (EP of communities) and phenotype diversity (EP of ecological functions and interactions). It would be of little use for our purpose to establish the catalogue of meanings found in the literature. Instead, we need an operational definition of EP to clarify what we expect from the objective of conserving it. Consequently, we define evolutionary potential as *the property of a biological entity (e.g. genome, trait, population, species, ecosystem) to be able to experience heritable change in some of its components between times t and t + *Δ*t*. This definition remains within the realm of evolutionary biology but avoids restricting our definition to genetic inheritance. We assume that if the knowledge of evolutionary mechanisms has a value for conservation, it should be true for different types of inheritance such as genetic, cultural or epigenetic. Moreover, we consider that higher‐level entities (e.g. a community) can experience heritable change through their components (e.g. species).

A second (and major) challenge is to clearly determine the role of evolutionary potential in conservation, that is *the prescriptions* one can derive from this concept. For example, should one preserve EP as a means to conserve entities in their present state (*biodiversity pattern*), or as a means to ensure the continuity of evolutionary processes (*biodiversity‐generating process*)? Some may argue that these aspects are two sides of the same medal so that making such a distinction is unnecessary. We are afraid that things are not that simple. We find a lack of formal consideration of the epistemological issues regarding the CEP (biodiversity‐pattern versus biodiversity‐generating process perspectives), and of a clear conceptual framework to position them with respect to their ultimate normative goals, that is: why EP should be conserved *in fine*? (Box 3). For example, different objectives, such as maintaining species richness versus ecosystem services, may be perceived as complementary in the conservation literature (e.g. Hoelzel et al., 2019) when they express different—when not conflicting—underlying values attributed to biodiversity, or prioritization of those values. We suspect that the recent and rapid development of conservation genomics (Harrisson et al., 2014; Ouborg, Pertoldi, Loeschcke, Bijlsma, & Hedrick, 2010) has contributed to accelerate the focus of scientists on “what” and “how” evolutionary potential (gene variants) needs to be conserved, leaving behind the fundamental questions of “why” and “when” it should be conserved (Kardos & Shafer, 2018).

Finally, while CEP is viewed as a *means* in the vast majority of instances, there is also a tendency by some to consider it as an *end* in itself, without any explicit qualification of the normative goal it is supposed to contribute to. In this case, conserving evolutionary potential is rather a purely technical end that should be distinguished from the nonanthropocentric, *process‐centred,* normative commitment to preserve evolution as a process in itself (Box 3). Consciously or not, this shift has probably been fed, at least in part, by the introduction of molecular genetics and genomics in conservation research.

**FIGURE 1:**
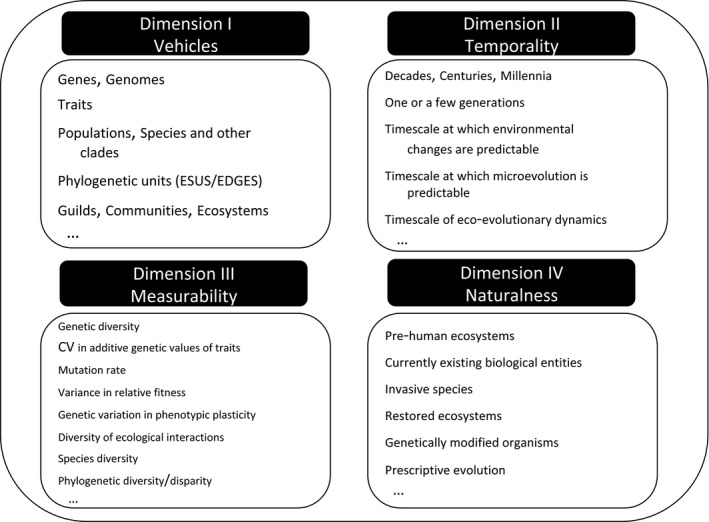
The four dimensions of evolutionary potential at a glance. Boxes contain examples of elements that can represent each dimension

Here, we contend that those who advocate for the conservation of evolutionary potential should explicit their epistemic and normative commitments by positioning their conception of CEP along four dimensions: (i) What are the vehicles of the evolutionary potential at stake (genes, traits, individuals, populations, communities, etc.)? (ii) What is the timescale at which evolutionary potential is considered? (iii) What is the measurability of evolutionary potential? (iv) What kind of human influence or intervention on evolutionary potential is compatible with their normative commitment? (Figure 1). These four dimensions relate to ontology (the “vehicles” or entities that instantiate evolutionary potential), scope (“temporality”), tractability (“measurability”) and naturalness (“human influence”). We need to address these four dimensions if we are to determine why, when and how the maintenance of evolutionary potential converges with the conservation of biodiversity, as well as with conservation values that benefit from wide societal acceptance.

BOX 1 **From Bernatchez's laboratory to the Mile End Club Social.**

Emmanuel Milot did his PhD in Louis Bernatchez's laboratory on the genetics of wandering albatrosses, quite an outlying study system for this fish‐oriented team. But Louis was not the kind of person to say no to a project that aimed to address stimulating questions in ecology or evolution; so he jumped into the project with enthusiasm. Early in the project, an unexpected obstruction arose when a leading senior researcher on seabirds, not a geneticist, decided to do a similar genetic study on the same bird populations, despite a previous agreement made to avoid overlap between projects. Milot later learned through the branches that this researcher was pushing his team to publish first. What a stressful situation for a starting PhD student! When Milot informed Bernatchez about the situation, he answered with much wisdom: “Don't worry. The most important is not the study system but the ideas. If you do come up with clever research questions, you'll do original research that will get published. Hey! How many of us, you think, are currently working on Atlantic salmon genetics?” Louis was right: in the end, Milot's PhD results were published *after* the other team but in higher impact journals. This is one of a few important lessons that Milot retained from Louis Bernatchez and that he now communicates to his own students.

It turned out that the albatross project showed that the birds apparently thrived with extremely low genetic diversity, an observation at odds with the prevailing conservation discourse. This observation raised interrogations about applications of genetics to conservation: Why, apart in clear cases of inbreeding depression, genetic information did not percolate more in management plans? Why some people obviously having a fixist perspective praised for the conservation of evolutionary potential? Why seemingly conflicting propositions in conservation biology were viewed as complementary? And what part of the ever‐growing attention given to genes was explained by the knowledge we gain about their true importance for conservation, versus by a form of reductionism that develops at the pace of technological advances, keeping our sight away from the big picture? Milot discussed his thoughts with Arnaud Béchet and Virginie Maris, two friends of his who are, respectively, a population biologist and a philosopher of biodiversity, both also long interested in conservation biology and quite critic of some mainstream ideas in the field. Taking advantage of their recent sabbatical leave in Montréal, they and Milot met periodically at Club Social, a small Italian café in the Mile End district of the city, to write the draft of this paper. The complementarity of their backgrounds made a good blend to tackle the issue of evolutionary potential with (they hope) a sufficiently broad perspective, although many questions they had still remained unanswered.

BOX 2 **Glossary**

*Ecosystem resilience*: The capacity of an ecosystem to respond to a disturbance by resisting damage, recovering quickly and returning back to its initial state.

*Epistemic intuition*: Set of knowledge, representations and beliefs that are embedded in a scientific theory or explanation.

*Evolutionary potential*: The property of a biological entity to be able to experience heritable change in some of its components between times *t* and *t* + Δ*t*. This entity can be for example a genome, a trait, a population, a species, an ecosystem, or something else.

*Expression of evolutionary potential*: The transformation of the evolutionary potential that initially exists at time *t* into heritable modifications of its vehicles. The most obvious example is the transformation, by natural selection, of variation into adaptation (to be distinguished from the *transfer* of evolutionary potential).

*Genetic essentialism*: A reductionist view whereby the identity of biological entities is in essence determined by the genes they carry. Genetic essentialists advocates for the restoration of pure genetic lines of species, which may have been hybridized for instance.

*Normative intuition*: Intuition on which we rely to justify which end of an action is good or bad.

*Normative value*: The value of an action judged morally right or wrong.

*Transfer of evolutionary potential*: The shift of evolutionary potential from one form or vehicle to another. For instance, hybridization can reduce the potential of two species to adapt to separate niches but increase that of an admixed population to colonize new habitats (should be distinguished from the *expression* of evolutionary potential).

*Vehicles of evolutionary potential*: The biological entities whose capacity to evolve is required for the realization of conservation objectives.

BOX 3 **What are the values at stake in the conservation of evolutionary potential (CEP)?**

**A. Anthropocentric values**

CEP is a mean for the long‐time supply of ecosystem services.

Faith et al., 2010: “Evolution continually produces new and often improved “solutions” as environmental circumstances change, and it thus has the capacity to provide new services and benefits (including new ecosystem services) to humans in perpetuity.”

**B. Nonanthropocentric entities‐centred values**

**B 1. Individual‐centred values**

The CEP of animal populations facing rapid environmental changes may be a vital asset to allow the survival and welfare of the individual members of these populations.

Wallach et al., 2018: “If the task of conservation is to actualize a human relationship with nonhuman nature that is sustainable and ethically appropriate, it is important that morally relevant individuals not be excluded from the scope of conservation concern. To this end, we contend that compassion is a critical element of ethically appropriate conservation practice.”

**B 2. Group or system‐centred values**

CEP is a way to preserve present biodiversity or specific biological entities (e.g. species, ecosystems) that are intrinsically valued.

**C. Nonanthropocentric process‐centred values**

CEP is an end in itself because the process of evolution is valued for itself. Biodiversity is a means for the future evolution of life.

Soulé, 1985 “…the continuity of evolutionary potential is good. Assuming that life itself is good, how can one maintain ethical neutrality about evolution? Life itself owes its existence and present diversity to the evolutionary process. Evolution is the machine, and life is its product. One possible corollary of this axiom is an ethical imperative to provide for the continuation of evolutionary processes in as many undisturbed natural habitats as possible.”

**D. Pluralistic values**

Conservation is targeting a plurality of values, both anthropocentric and nonanthropocentric, entities‐centred and process‐centred (e.g. species AND evolutionary potential). Biodiversity and evolution are thus two complementary goals of conservation that are both instrumentally and intrinsically valued.

Erwin, 1991: “The goal of conservation strategy should be the protection of future maximum biodiversity as well as the preservation of contemporary species of human interest.”

## 2 DIMENSION I: THE VEHICLES OF EVOLUTIONARY POTENTIAL

To reconcile the apparent contradiction between “to conserve” and “to let evolve” we need to define what entities—the vehicles—should be mobile along their evolutionary trajectory in order to keep other entities or properties in a desirable state. Fundamentally, this is not only a scientific question because the answer will depend on our ethical conception of what entity should deserve our moral attention and thus be conserved or prioritized (Brooks et al., 2006; Maris, 2016; Redford et al., 2003; Box 3). Consequently, we define the **vehicles of evolutionary potential** as *the biological entities whose capacity to evolve is required for the realization of conservation objectives*. Therefore, in this context evolution should be understood as any heritable change in a vehicle between times *t* and *t* + Δ*t*.

Identifying the vehicles relevant to conservation objectives is not necessarily straightforward. Consider, for instance, the goal to conserve evolutionary potential such that all taxa living in a given area survive as identifiable distinct entities over the long run. Such a valuation of biodiversity pattern underlies many management plans targeting particular areas or species, as well as policies and legal frameworks governing these plans, such as the US Endangered Species Act or the European Union Bird Directive. A first step is often to delineate the evolutionary significant units (ESUs) to be considered. ESUs represent monophyletic groups constituted by individuals sharing genetic ancestry and ecological characteristics that may represent significant adaptive variation within a species (or species themselves), ideally based on concordance of data derived from different techniques (Ryder, 1986). The search for hidden ESUs is often considered a key requisite in conservation planning as it serves to define conservation units (CUs; Funk, McKay, Hohenlohe, & Allendorf, 2012). In some countries, ESUs are the legal entities on which conservation policies are implemented.

However, the link between ESUs and CEP is not always straightforward because the vehicles of evolutionary potential at stake can vary as a function of conservation values and objectives. When the goal is to preserve ESUs themselves, quantitative change, such as in the mean of a phenotypic character, may be more welcomed than ontological change, such as the genetic modification of species through hybridization. Hence, the appropriate vehicles may not be ESUs but perhaps a subset of phenotypic traits. Alternatively, ESUs could be the vehicles relevant to maintain the diversification process that generates the diversity of communities and ecosystems. Crucially, the relevant EP vehicles can differ as a function of the level(s) of biological organization that is (are) targeted by conservation priorities, leading to opposite prescriptions. The problem can be best illustrated with a concrete example that underpins the dilemma and tension that can exist over conservation norms.

### 2.1 What goal? What vehicles? An example on warblers

The example is about a pair of hybridizing bird species that represent a particularly tough case for conservationists. The blue‐winged warbler (*Vermivora cyanoptera*) and the golden‐winged warbler (*V. chrysoptera*) are two closely related North American passerines. Formerly, their geographic ranges did not overlap but they do now as a result of landscape modifications in the past century. Where this occurs, the two species hybridize without apparent fitness consequences for reproducing individuals, including hybrids and backcrosses (Vallender, Friesen, & Robertson, 2007). Nonetheless, hybridization favours blue‐winged genes, partly because females of both species prefer golden‐winged mates, causing the disappearance of pure golden‐winged phenotypes after a few decades of contact in a given area (Gill, 1997).

Saving golden‐winged warbler populations from this process of “genetic dilution” has been considered a critical issue (Vallender & Bull, 2016). Yet, contrasting opinions are possible with respect to CEP. From a *biodiversity‐pattern* perspective, the loss of either warbler species may not be acceptable. Consequently, we may wish to maintain EP within both species as a means to ensure their viability as separate entities. Conserving more species might also provide insurance to the community against environmental stochasticity and future environmental changes. From a *biodiversity‐generating process* perspective, the reproductive interplay of the two species could be seen as the **expression of the evolutionary potential** of the blue‐/golden‐winged system taken as a whole, thereby responding to human‐induced environmental change. Arguably, this could also be a mean of maintaining biodiversity, perhaps not under the form of two separate species but as a merged one adapting to its new environment (a possibility echoed by a recent genetic study putting into question the species status of the two warblers; Toews et al., 2016). Therefore, from the *biodiversity‐pattern* perspective hybridization could be viewed as a loss because the relevant EP vehicles are not the species themselves, but perhaps their populations or traits (see also the example in Box 4). From the *biodiversity‐generating process* perspective, hybridization could be considered as the expression of the evolutionary potential at a higher level of biological organization. For instance, if our goal is to conserve a community of species exploiting the various resources and niches of a habitat, we might believe that hybridization between closely related populations is, in certain cases, a better way to achieve this goal than attempting to save the integrity of each separate species. Under this scenario, the number and identity of species (vehicles) in the community can change with time. Therefore, both perspectives are likely to lead to drastically different prescriptions regarding whether or not one should prevent hybridization in order to conserve biodiversity. Garrick et al. (2014) bring a related argument about the (natural) hybridization between two intraspecific lineages of the Galápagos giant turtle *Chelonoidis becki*. They suggest that lineage fusion could sometimes be beneficial to maintain or increase EP.

BOX 4 **Evaluating evolutionary potential in the spadefoot toad**

The spadefoot toad (*Pelobates cultripedes*) is a near‐threatened amphibian with a patchy range in Spain and southern France. An effort is presently made to assess the potential of adaptation of this species to climate change. This goal could stem from both nonanthropocentric (group‐centred) or anthropocentric (e.g. the opportunity to observe and enjoy fauna being a service provided by the ecosystem) normative values. Using genomic data on thousands of loci, it will be possible to search for genes involved in the adaptation to temperature, humidity and hydroperiod of ponds. In particular, this will be done by searching whether the populations in the northern part of the range, where a gradual aridification of the climate is expected over the next 50 years, share alleles with southern populations that help the latter to tolerate arid conditions. Results will help understanding the genetic mechanisms associated with adaptation to local climatic conditions. They may provide a basis for managers and decision‐makers to anticipate the effects of climate change on the conservation and management of this species. Here, the vehicles are identified as both populations and traits: if the northern population appears impoverished in alleles adapted to aridification, then management reinforcing northern populations with representatives of southern ones will be implemented to improve their evolutionary potential and secure adaptive traits in the future. Therefore, it is admitted that both traits and populations can change to the extent that a recognizable spadefoot toad species is maintained. In this case, the delineation and lineage specificity of populations are likely to be blurred by translocation and subsequent hybridization.

### 2.2 Is genetic diversity a relevant indicator of evolutionary potential?

The warbler case underscores the need for conservation biologists to make explicit their target vehicles when they evoke EP, in order to unveil the conservation values motivating their work, as well as to justify management choices. Clarification is particularly important when the emphasis is on genetic diversity (*sensu* *lato*) as an indicator of EP within a population. In a kind of extension of Dawkins (1976), who defined the organisms as the vehicles of replicators (the genes), the population of organisms is typically considered as the vehicle of EP (diversity of replicators), although the idea is generally not expressed in these words. However, we must question when this vehicle is the relevant one to focus on. For instance, a given level of genetic diversity may represent a potential for evolutionary changes greater than one is ready to accept. Imagine that alleles giving a dark colour to an emblematic species like the polar bear spread within the whole species, due to some fitness advantage under a warming climate; or that some genetic variants allowed a naturally occurring predator to evolve phenotypic changes that would make it predate (and depend) on an invasive species, itself the target of eradication efforts (this example also raises the issue of naturalness—discussed in Dimension IV). Would these components of genetic diversity represent an evolutionary potential that matches conservation values?

Finally, a vehicle needs not pertain to a single level of biodiversity. Those vehicles at a lower level of organization contribute to the EP at higher levels. For example, the evolution of a given trait in a species can modify ecological interactions, such that the EP of this trait also translates into the EP of ecosystem attributes (e.g. predator–prey coevolution in fish in Trinidad was shown to influence ecosystem‐level parameters, such as algal biomass; Palkovacs et al., 2009). There is probably no easy recipe, applicable to all cases, to identify the relevant EP vehicles (see examples in Table 1). Nevertheless, we believe that an important step for meaningful conservation relying on evolutionary potential should be to separate as clearly as possible the vehicles that can evolve from the biological entities or properties we hope to preserve. It seems useful to start by considering as potential vehicles the entities that lie, on the scale of biological organization, just below those entities that are priority conservation targets (Table 1). Identifying the relevant vehicle for the conservation issue at stake should also help to determine the appropriate measure of EP to use (Dimension III), as well as identifying collateral impacts on other biological entities which can themselves be the relevant vehicles for other conservation goals.

**TABLE 1:** The four dimensions of evolutionary potential. This table presents a few examples of (hypothetical) CEP proposals, along with information and considerations for each dimension. Note that for each proposal, the elements given under the four dimensions may vary depending on the specific details of the proposal or underlying normative values. We insist here that the brevity of these elements, simply given as examples, should in no case be interpreted as if a few words or points are always sufficient to account for these dimensions; thinking about temporality, for instance, may necessitate considering many different aspects

Target	Proposal	I. Vehicles	II. Temporality	III. Measurability	IV. Naturalness	Example of conflict with alternative proposition
Maintain locally adapted populations within species or ESUs	Actions to conserve local genetic diversity at ecologically important traits	Phenotypic traits	Decades to centuries, or variable according to the timescale of anticipated environmental changes	Coefficient of variation in additive genetic values; functional SNP variation; must consider the difficulty to predict genetic responses to selection	Does not admit substantial changes in the current biodiversity having evolved in natural habitats	Opposes to population admixture helping adaptation to future conditions at the expense of losing some local adaptation to current environments
Maintain diversified communities in human‐altered landscapes	Actions to conserve a diversity of habitats and ecological interactions in altered landscapes	Species, guilds or communities themselves	Centuries	Species diversity; habitat complexity; diversity of phenotypic functions	Accepts some human‐induced modifications to communities	May admit that new species colonize an area, or hybridization between species, following human disturbance, to the detriment of species “integrity”
Maintain metacommunities, global biodiversity	Adaptive networks^a^	Communities, ecosystems and their components	A few generations or decades (presumably)	Composite EP measure (see §3.1); feasibility and temporal tractability need to be demonstrated	Does not focus solely on pristine ecosystems or attempt to restore ecosystems to prehuman states, accepts human interventions to improve habitats	Opposes to restoration of historical communities
Maintain a provisioning service in forestry (timber production)	Assisted migration of tree species adapted to warmer climates	Forest ecosystem	Decades	Variance in primary productivity within and among species	Low level of naturalness	Opposes a reduction in the production of biomass, due to global warming, in forests which can otherwise provide new refuges for free evolution without exploitation of resources

^a^ see Webster et al., 2017

## 3 DIMENSION II: THE TEMPORAL DIMENSION OF EVOLUTIONARY POTENTIAL

Evolutionary potential refers to the potential for a change, and change cannot be expressed without the passage of time. Despite its importance, the temporality underlying CEP is rarely discussed explicitly or at length (but see Mace & Purvis, 2008). If CEP is desired, there should be the potential for a change at a temporal scale consistent with conservation objectives (hence our EP definition as *the property of a biological entity to be able to experience heritable change in some of its components between times t and t + *Δ*t*). Consequently, the relevant EP to consider at time *t* depends on the state of the system that is desired at (or at least up to) time *t* + Δ*t*, where Δ*t* could be as short as a few years or as long as centuries or more, depending on the objective at stake. Thus, a key question is as follows: Can we predict evolutionary change at the relevant timescale and realistically undertake actions that could be effective over that timescale?

### 3.1 *Short‐term* versus* long‐term evolution*

The natural fate of species (speciation, evolution, extinction) has to do with long, macroevolutionary time, typically millennia or more. Advocates of evolutionary conservation may have a shorter temporality in mind, more in tune with human action. In addition, the positive impact of CEP may be more predictable when both environmental changes and appropriate responses of natural populations (e.g. range shifts necessitating the evolution of dispersal capability) can be anticipated, which is easier for shorter timescales. Yet, if we can easily anticipate, for example, that the climate will warm, then a strategy other than CEP could be to favour individuals with traits that confer higher fitness under warmer conditions (the “winners,” sensu Webster et al., 2017), thereby potentially reducing the genetic variance in those traits (assuming that the temporal scale targeted by conservation efforts is short, such as the number of generations corresponding to the timescale on which we can anticipate environmental changes with reasonable confidence). Therefore, we may be faced by the paradox that conserving EP impedes short‐term conservation of, say, populations when genetic variance is used to measure EP. Conversely, if future environmental conditions are hardly predictable, or expected to be highly fluctuating in space and time, then a good strategy might be to conserve today a larger variance in traits exhibiting a relationship with fitness that depends on climate (perhaps including phenotypes that are presently maladaptive), in particular if reaction norms differ across genotypes.

In a similar vein, Webster et al. (2017) propose the more holistic approach of “adaptation networks” to tackle uncertainty around future environmental conditions, which, they insist, is poorly addressed by the “predict‐and‐prescribe paradigm whereby conservation priorities are based on predictors of the responses of species and communities to projected future environmental conditions.” They emphasize that a “pick the winners” strategy, which consists in favouring the genotypes and phenotypes anticipated to do best in the future, is risky considering our limited capacity to predict the future, namely to assess the likelihood of each of the many potential outcomes. Seen under our 4‐dimensional framework, their proposal relies on a composite measurement of EP, integrating the maintenance of a diversity of biological options (genes, phenotypes, communities, etc.), the scale and level of connectivity among habitats, and the extent to which the structuring of metapopulations can buffer ecological risks. Here, the vehicles are whole ecosystems or communities, as the authors recognize that “adaptation network explicitly assumes that some aspects of biological diversity will be lost” and that “although individual communities or system elements might be extirpated, the metacommunity and the overall system remain viable” (Webster et al., 2017). While these authors do not explicitly discuss the temporal dimension, a relatively short timescale appears to be involved because their proposal aims to be an alternative to the actions undertaken under the predict‐and‐prescribe paradigm; these actions are usually set on short timescales (a few generations or decades).

In any case, conservation norms should not be subordinated to the temporal tractability of EP. It may be that CEP is desirable but hard to achieve. For instance, Robert et al. (2017) state that biodiversity conservation has to do with macroevolutionary temporality, which should not be conflated with the temporality of conservation actions because the two are immeasurable. They nevertheless add that maintaining the evolutionary potential of populations or species is perhaps the only scale at which we can act efficiently, while expecting this will help to preserve biodiversity over the long run (but see below).

Studies conducted in the last two decades show that evolutionary changes within populations or species occur regularly at timescales that coincide with management actions (Kinnison & Hairston, 2007), especially in highly anthropized habitats (Szulkin, Munshi‐South, & Charmantier, 2020), and may even influence ecological processes through eco‐evolutionary dynamics (Hendry, 2017). Therefore, microevolution of quantitative characters is amenable to some form of prediction (but see Morrissey et al., 2012). For instance, the coefficient of variation in additive genetic values (Houle, 1992) in breeding phenology, such as laying date in birds, may help predict whether or not populations are likely to genetically track environmental changes brought by global warming (Gienapp, Teplitsky, Alho, Mills, & Merila, 2008). In a few situations, the direction of future genetic changes after human interventions is even easy to predict. A well‐documented case is overharvesting of fish, which has led to a genetic decline in the size at maturity of several species (Sharpe & Hendry, 2009), a trend that can be slowed down or reversed with a reduction of stock exploitation. These situations may offer some leverage to conservation under Robert et al.’s (2017) hypothesis that short‐term CEP may help maintain biodiversity over longer term. However, there is still huge uncertainty about the impact this will really have. For example, the negative scaling of natural evolutionary rates with time (Kinnison & Hendry, 2001), or the specificity of forthcoming human‐induced evolutionary processes (Smith, Kinnison, Strauss, Fuller, & Carroll, 2014), could mean that the present or near future does not guarantee what the more distant future will be made of. Therefore, it appears critical to address the temporal dimension of EP, especially when we consider the financial, time and highly qualified human resources required to nourish evolutionary conservation with research.

### 3.2 The dynamics of evolutionary potential

Another aspect that deserves attention is the temporal dynamics of evolutionary potential itself, that is, beyond a static point of reference at time *t*. EP may change between time *t* and *t* + Δ*t*, which can contribute to, or impede, the fulfilment of conservation goals. In other words, we must separate the *existence* from both the *expression* and the **transfer of evolutionary potential** (see below), a distinction to our knowledge rarely made explicitly.

If EP were never expressed, then there would be no point to conserve it. Consequently, conserving EP also implies accepting its erosion (e.g. by natural selection) or its expression (notwithstanding that evolutionary potential can itself evolve; Jones, Arnold, & Burger, 2007; Kokko et al., 2017). A short‐sighted focus on genetic diversity at time *t* may restrain EP expression, hence the very purpose of evolutionary conservation, for example by protecting unfit phenotypes when efforts are made to maximize genetic diversity. Imagine that among a set of subpopulations, one of them has a lower diversity because it is adapting more quickly, through selection, than others. Thus, attempts to locally reintroduce genetic diversity would counteract the expression of adaptive EP. By contrast, alleles that are neutral at time *t* could later become adaptive because some fitness effects emerge only under specific environmental conditions, such as alleles conferring infectious disease resistance, which can be neutral when the pathogen is absent, or those associated with inbreeding depression. Even though variation at functional genes, whether currently neutral or not, could be targeted by CEP, we are still far from having an operational framework that can globally canalize conservation genetics into pipelines with robust predictable outcomes.

Evolutionary potential can also be transferred from one vehicle to another during the course of evolutionary history. EP existed in the very first nucleic acids on Earth, regardless of their low diversity. But the emergence of the different life forms known today required the *transfer* of evolutionary potential from a basic chemical potentiality to a form, genetic variation, that natural selection could feed on for its expression. This involved a (partial) transfer of EP from one level, biomolecules, to another level, populations of biomolecules, and, eventually, to cells, traits, and organisms carrying them. Likewise, a transfer occurs when blue‐winged and golden‐winged warblers hybridize, or when the arrival of an invasive species increases EP of a community while reducing that of some native species.

In summary, to (try to) set conservation objectives, it is not sufficient to limit our conception of EP to some measurable quantity (e.g. genetic diversity) at time *t*. CEP implies dialectic between the potential (existence) and the process (expression and transfer). Thus, ecologists should make explicit not only what evolutionary potential they aim to conserve, but also how, in practice, they integrate the dynamic nature of EP in their research or actions.

### 3.3 The reversal of temporality and the road to genetic essentialism

A radically different posture proposes to reverse temporality outright, through the restoration of the past genetic composition of populations or species. Examples include attempts to purify “wild” genomes from recent admixture with domestic breeds or to recreate extinct genetic stocks. An example of the former is the introgression of cattle genes in American bison populations (Derr et al., 2008). In such a case, genetic restoration could be useful if it targets functional variants, the loss of which having a documented impact on fitness‐related traits. Its usefulness is less clear when the proposal is to wipe out from wild populations any introgressed variant, for the sake of putatively restoring/maintaining adaptation and evolutionary potential. Both types of justifications have been advanced for the bison case (e.g. Derr et al., 2008; Freese et al., 2007). While they are usually based on established scientific principles, they do not always appropriately link CEP to conservation, namely: What EP are we talking about and does it relate to vehicles that are relevant to conservation objectives?

The risk here is to slide towards **genetic essentialism**, for which the recovery of “pure” historical genomes becomes an end in itself. An interesting example is the desire to recreate old lineages in Galápagos giant tortoises. Tortoises from Floreana Island, *Chelonoidis niger*, made a unique genetic stock that is now extinct. However, some of their offspring are still alive, being hybrids born to one parent from Floreana and one from another island, as a result of translocations over past centuries, at times when tortoises were exploited (Poulakakis et al., 2008). A campaign to find and identify these hybrids has taken place with the purpose to use them in sophisticated breeding designs to recreate the Floreana lineage, as purely as possible (Miller et al., 2017). Seemingly, such essentialism denies the role of evolutionary potential in conservation. At least, both the existence of EP and its eventual expression are not obvious under that perspective. Interestingly, conflicts over conservation objectives targeting Galápagos giant tortoises were recently discussed by Hunter et al. (2019), who developed a decision model showing that “timely, cost‐effective solutions can be identified in cases where management objectives appear to be incompatible.” This example is quite relevant for our purpose as it illustrates how important it is to identify when different proposals appear to be grounded in similar values or priorities while in fact they do not, or *a contrario* when proposals apparently divergent do serve the same values or priorities.

## 4 DIMENSION III: THE MEASURABILITY OF EVOLUTIONARY POTENTIAL

The will to conserve evolutionary potential implies the capacity to identify which EP it will be relevant to conserve under hypothetical scenarios of environmental change. In addition, in Dimension II we mentioned the shortcomings of limiting EP evaluation to a measurable quantity at time *t*, without accounting for its temporal dynamics. Consequently, an additional burden rests on scientists who should also demonstrate that the way they measure EP (a) pertains to the appropriate vehicles; (b) conveys knowledge that is useful to inform conservation actions and policies.

This questioning is particularly important with the advent of the genomic era, which brings an unprecedented wealth of data on variation at all types of loci across whole genomes (coding, noncoding, functional, neutral loci) and the mapping of this variation to phenotypes. It is now possible to model genomic data from a species across landscapes and map it to adaptation, like we model communities to identify species adapted to different environmental conditions. This includes the prediction of differences in the genetic adaptation of populations under current and future climates (Fitzpatrick & Keller, 2015; Box 4). Bay et al. (2018) proposed “the genomic vulnerability” metric, which measures “the mismatch between current and predicted future genomic variation based on genotype‐environment relationships modeled across contemporary populations,” to identify populations likely to be most vulnerable to climate changes. Fitzpatrick, Keller, and Lotterhos (2018) question this approach (even though the “genetic vulnerability” concept in Bay et al. is similar to the “genomic offset” metric they themselves developed; see Fitzpatrick & Keller, 2015). They state that “Further testing and validation are needed to verify the extent to which genetic offsets reflect changes in fitness expected in new environments […] before genetic offsets can be considered ‘an important tool for making more‐informed conservation decisions’…” (Fitzpatrick et al., 2018). If genomic tools may represent a capacity never reached before to measure evolutionary potential, they also increase the risk of practicing a discovery‐oriented conservation that loses sight of the norms motivating actions in the first place. Such inflation of the value of genetic data had already begun with studies based solely on few genetic markers (Reed, 2010). As underscored by Kardos and Shafer (2018), a peril of discovery‐oriented conservation is to spend a considerable amount of resources that could be better used otherwise.

### 4.1 Nonmolecular indicators of evolutionary potential

The assessment of evolutionary potential is not limited to molecular metrics. Life history traits and behavioural characteristics, assuming they are heritable, have been proposed as EP predictors. One example is the position along the slow–fast life history continuum: species with high fecundity and low survival were found to be more affected by climate change than others (Jiguet et al., 2007; see also the review by Sepp, McGraw, Kaasik, & Giraudeau, 2018 on the link between urbanization and the pace of life). Another example is dispersal, which facilitates gene flow among isolated populations and can mitigate the deleterious effects of inbreeding. Actually, species with high natal dispersal do better at facing climate changes than those with lower dispersal rates (Jiguet et al., 2007). This is because dispersers have better opportunities to track habitat changes and optimal environmental conditions. For the same reason, connectivity between populations can be an indicator of EP (Crooks & Sanjayan, 2006; Ladle & Whittaker, 2011; Webster et al., 2017) even though gene flow can hamper local adaptation (Bridle, Polechová, Kawata, & Butlin, 2010).

At another biological level, calls for rewilding disturbed ecosystems rely on the assumption that by increasing functional diversity, large herbivores, carnivores or other megafauna will contribute to the **resilience** **of ecosystems** to global change (Donlan et al., 2005; Sarrazin & Lecomte, 2016). Indeed, ecosystems impoverished by anthropogenic disturbance are less resilient and more prone to ecological tipping points, whereby systems shift radically and potentially irreversibly into a different state (Díaz & Cabido, 2001). The aim to maintain the resilience of an ecosystem, in which case lower biological entities that constitute it (e.g. species) become the EP vehicles, may oppose to the aim to conserve its evolutionary potential, whereby the ecosystem itself is the vehicle linked to other targets (e.g. maintain ecosystem services).

### 4.2 ESUs versus the phylogenetic history perspective

ESUs have long been used as targets of conservation. A different yet connected perspective is the conservation of phylogenetic diversity, informed by combining phylogenetic data and threat levels in an approach called EDGE, for “evolutionary distinct globally endangered entities” (Isaac, Turvey, Collen, Waterman, & Baillie, 2007). Its goal is to temper the propensity to unduly increase the number of “new” threatened and endemic species resulting from the application of phylogenetic species concepts. Indeed, under the EDGE approach any increase in extinction risk due to taxonomical splitting is somewhat balanced by a decrease in evolutionary distinctiveness. Beyond preserving evolutionary history, this approach fits the objective of increasing the evolutionary potential of a community (the vehicle considered here). The justification is based on a general insurance hypothesis: maximizing the representation of phylogenetic history, rather than the number of species, increases the chances to maintain the best evolutionary options for the future of ecosystems (Erwin, 2008). This approach has for instance been defended to prioritize the conservation of the incredible flora diversity of the Cape region in South Africa (Forest et al., 2007).

Some propose to infer the evolutionary potential of lineages from the topology (e.g. branch lengths) of phylogenetic trees. Species with few close relatives (i.e. high evolutionary distinctiveness) may be “relicts” or “living fossils” with limited potential to generate new diversity, while short branches resulting from recent radiations may have higher evolutionary potential (Erwin, 1991; Mace, Gittleman, & Purvis, 2003). Therefore, families with short phylogenetic branches may better cope with environmental changes than deeper lineages with only one or few threatened species having little chance, if any, to produce other species in future speciation events (Erwin, 1991). Following these arguments, to maintain EP one would bet on protecting common species from lineages characterized by many speciation events. Yet, there is currently no theory predicting which lineages will speciate in the future, or where the next adaptive radiations will come from (Krajewski, 1991) and the timescale involved. Moreover, phylogenetic trees are not living entities. The fate of a given lineage is dependent on the species composing that lineage, species‐specific features shaping their vulnerability, including genetic structure, life history, and behaviour, along with population size and geographic range. These dynamics traits cannot be easily deduced from a phylogenetic tree.

Stanton et al. (2019) question the idea that the best way to conserve the evolutionary potential of populations (and their genetic components) will be by choosing the appropriate approach to delineate taxa of interest (ESU versus EDGE approach). Instead, they praise for a more pragmatic approach using genomic tools to characterize adaptive potential, regardless of species concepts, or even without invoking a species concept at all. Such proposals would benefit from the explicit identification of the EP vehicles they imply, hence of the underlying normative commitment. In other words, pragmatism is likely to be a quality in any conservation action, but pragmatism for what?

### 4.3 Hybridization and evolutionary potential

A challenging issue regarding the measurability of evolutionary potential stems from cases of hybridization, which can follow different pathways. In some cases, hybridization transfers an evolutionary potential that may then be expressed by the exploitation of a new niche. For example, a new species of Darwin finch in the Galápagos emerged from only three generations of interbreeding between *Geospiza fortis* and *G. conirostris* (Lamichhaney et al., 2018). In other cases, exotic species come into contact and interbreed with closely related native species, changing the evolutionary dynamics. Alteration of habitats fosters reconnection of ecologically segregated species, facilitating hybridization, and create novel conditions in which hybrids may thrive. Many opportunities arise for hybridization between domestic and wild species as well. For example, crop‐related hybrid weeds can cause considerable agricultural losses and rapidly gain advantageous genes from their domestic relatives, e.g. conferring herbicide resistance (Pandolfo et al., 2016). Climate change may alter species ranges and trigger hybridization between previously isolated species (Brennan et al., 2014). Hybridization can restore heterosis or alleviate inbreeding depression. For instance, controlled hybridization of the rare Florida panther *Puma concolor coryi* with the related Texas cougar *P. c. stanleyana* increased genetic diversity in the former, leading to a rapid demographic recovery (Hedrick, 1995). Alleviation of locally expressed inbreeding depression occurs also naturally, such as when genes from song sparrows (*Melospiza melodia*) from mainland British Columbia are introduced into the inbred population living on the nearby Mandarte Island (Keller, Arcese, Smith, Hochachka, & Stearns, 1994). More generally, vonHoldt et al. (2018) discuss the need to take into account, in conservation plans, the role played by hybridization and admixture in speciation and the diversification of life. Undoubtedly, these processes have had a major impact on the varied forms that the potential for evolution takes.

### 4.4 Asking the right questions to identify the appropriate EP metrics

As we have seen, different scenarios of hybridization necessarily translate into various forms and degrees of EP transfer, which may or may not support conservation objectives. This raises the more general question: How appropriate are EP indicators like ESUs‐related or other metrics to meet conservation objectives? Again, we must not lose sight of the goal pursued. Among possible ecological or evolutionary outcomes in the future, some are more desirable than others in terms of conservation values at stake. Research may want to evaluate the probability of different outcomes to guide management decisions, policies and resource allocation. The latter point can be conveniently expressed in Bayesian language: given a prior opinion about the probability of different evolutionary outcomes at time *t* + Δ*t*—some being preferred to others—what additional scientific knowledge gathered about EP can update this opinion, or at least reduce the uncertainty around it? Asking this question can help assess what EP measurement is most relevant for conservation purposes.

Finally, in Dimension II we distinguished the *existence* from the *expression* of evolutionary potential. Thus, to assess the effectiveness of the CEP in achieving conservation objectives, it seems logical that the measurement of EP should be accompanied by monitoring of current/future evolution fueled by this potential (one example is the temporal monitoring of adaptive alleles in a population, but see Kardos and Shafer (2018) for the limits of this approach). In any case, we should keep in mind that monitoring EP, even with appropriate metrics, will be of limited value when conservation actions can have no traction on it, or otherwise at prohibitive costs in resources.

## 5 DIMENSION IV: NATURALNESS AND THE CONSERVATION OF EVOLUTIONARY POTENTIAL

The last dimension we explore is about the relationship between human influence and the conservation of evolutionary potential. We introduce it with another example of human‐facilitated hybridization. Native to North America, the ruddy duck (*Oxyura jamaicensis*) now occurs in 21 Western Palearctic countries after its accidental introduction in this region (Hughes et al., 2006). Mitochondrial DNA analysis has shown that European ruddy ducks likely derive from a group of solely seven birds escaped from captive stocks in the UK (Munoz‐Fuentes, Green, Sorenson, Negro, & Vila, 2006). In contrast, the white‐headed duck (*O. leucocephala*), which is native to the Palearctic, has undergone a considerable decline in range and population owing to habitat degradation and hunting. Where they meet, the two species freely hybridize and hybrids are fertile, threatening the integrity of the white‐headed duck. Humans bear responsibility in the hybridization of ducks, as they do in the warbler case discussed earlier. This responsibility is arguably more direct in the duck case since hybridization was initiated by (involuntary) artificial introduction of ruddy ducks on a new continent. This probably explains why the actions proposed to limit the consequences of hybridization are more drastic for ducks—targeting, for example, the eradication of ruddy ducks in certain countries (Hughes et al., 2006)—than for warblers, such as landscape management (Rohrbaugh et al., 2016). Hybridization seems to be more tolerated by conservationists when humans are not responsible for it (vonHoldt, Brzeski, Wilcove, & Rutledge, 2018) (interestingly, the control of ruddy ducks is controversial as this species is welcomed as a nice addition to European avifauna by some people). This raises the broader issue of the degree of naturalness that underlies any conservation vision.

During the past decade, the field of conservation biology has been shaken by the heated quarrels opposing tenants of traditional conservation to those of the so‐called new conservation movement (Kareiva & Marvier, 2012). To make a long story short, the cornerstone of the debate is the commitment of traditional conservation to a certain value of naturalness or wildness. Indeed, two ideas widely shared among conservationists (Takacs, 1996) are challenged by “new conservationists”: (a) nature and biodiversity should be valued for themselves, as proper ends and not as means of extrinsic ends such as human benefits (Soulé, 1985; Vucetich, Bruskotter, & Nelson, 2015); and (b) human influence is of a different kind than nonhuman influence and this difference should count in the attribution of conservation values (Angermeier, 2000).

Curiously, those engaged in the conservation of the evolutionary potential have apparently not engaged very far in this debate (apart, perhaps, proposals approaching genetic essentialism). Yet, the naturalness issue is particularly acute here because CEP projects us in a future where biodiversity not only is preserved, but also evolves into new states that can approach or drift away from “natural” reference points. Moreover, naturalness can refer either to the expected state of biological entities targeted by CEP or to the degree to which the evolutionary potential itself is “natural” versus aided by (or the result of) human interventions. For instance, the translocation, from one population to another one of the same species, of individuals carrying alleles presumably better adapted to future environmental conditions, is clearly a human manipulation of EP, even if the goal is to maintain a species over its historical (natural) range. Thus, the fit of CEP to the degree of naturalness underlying conservation values and goals must be looked at from the broader perspective of normative issues surrounding the conservation of biodiversity.

### 5.1 Anthropocentric versus nonanthropocentric perspectives

As we have seen, justifications for biodiversity conservation can rest on different normative frameworks (Box 3). Conservation can be justified for the sake of human beings, an old rationale (e.g. Pinchot, 1910) that is reviving with the “ecosystem services” perspective (Mace, Norris, & Fitter, 2012). Under this anthropocentric view, our responsibilities towards natural entities are more indirect, more rooted in human interests. Within the context of global environmental changes, the evolutionary potential of ecosystems to adapt to new environmental conditions could be a key feature to maintain or enhance the provision of ecosystem services (here, ecosystems would be the vehicles and the services they provide the target of conservation). Furthermore, the evolutionary process itself may provide benefits to humans, coined “evosystem services”; some authors go as far as to metaphorically qualify the evolutionary process a “factory for human uses” (Faith et al., 2010, p.69), for instance when a native species evolves rapidly to predate a harmful exotic species.

Another set of justifications for conservation groups nonanthropocentric values, which do not specifically target human interests, such as their consumption, well‐being and rights, but nonhuman entities instead. From a biocentric perspective (Box 3), it is the living organisms (individuals) that are valued for themselves, a perspective rarely evoked in conservation, though. The field of “compassionate conservation” is committed to take into account the interests and well‐being of sentient animals in conservation practices, opening an avenue for the respect of individual beings rather than only that of supra‐entities, such as species or ecosystems (Bekoff, 2013). However, it is unclear how CEP can be instrumentally valuable for an individual organism. A biocentric perspective might consider that for individuals confronted to changing environments, being able to express adaptive traits is beneficial if it means less stress or suffering for them.

A nonanthropocentric normative commitment that is more consensual among conservationists is to value collective entities, such as populations, species or ecosystems. Thus, CEP is viewed from a *biodiversity‐pattern* perspective as a way to preserve current biodiversity or specific biological entities that are intrinsically valued. Alternatively, some authors in environmental ethics and in conservation biology defend the idea that it is the processes of life, not just existing entities, that should be valued for themselves, and among these processes, the evolution of life is a prominent candidate for intrinsic value. From this *biodiversity‐generating process* perspective, evolutionary potential, in almost any form, becomes an end for conservation as much as a means, and CEP is a critical way to preserve life‐continuing evolution (Box 3).

### 5.2 The nature of human influence

The second idea widely shared among traditional conservationists, i.e. that human and nonhuman influences are different in nature, is obviously about naturalness. Many will agree that the free evolution of two lineages after a continental drift is more “natural” than the directed evolution of adapted varieties of crops. The normative intuition motivating CEP will not be the same depending on whether we target/accept “natural” evolution, “artificial” evolution, or both, and likewise for natural versus human‐created evolutionary potential. At one end of the spectrum, one way to conserve evolutionary potential would be to do nothing and let nature evolve freely. After all, the potential for the evolution of all life forms existed in the first nucleic acids. This argument could be made in praise of CEP both as a means, for instance to allow life to adapt to urbanized environments with no concern for existing biodiversity, and as an end, for example if one valued any kind of unassisted evolutionary change over conservation. Interestingly, we see by this example that favouring the “natural” (unassisted) expression of evolutionary potential does not necessarily imply the conservation of a more pristine, or “natural” state of biodiversity.

At the opposite end of the spectrum, prescriptive evolution was defined as the “use of planned manipulations of evolutionary processes to achieve conservation outcomes [...]” (Smith et al., 2014). For example, one may favour individuals with (genetic values for) phenotypes that are expected to be better adapted to changing environments (an issue already discussed in Dimension II). Steering evolution can even fall outside the scope of CEP, for instance the editing of genomes with CRISPR‐Cas9 to introduce (and even create) better‐adapted alleles in wild populations. Here, the requirement of evolutionary potential is bypassed: instead of maintaining/creating EP, adapted organisms are directly constructed. However, such high‐tech approaches have more to do with bio‐engineering than nature protection. Despite a recent IUCN report discussing the potential contribution of synthetic biology to nature protection (Redford, Brooks, Macfarlane, & Adams, 2019), they should not be conflated with conservation.

As we see, when CEP is justified on an anthropocentric basis, the level of human influence interferes less with conservation values. What matters is the capacity of ecosystems to deliver services (*sensu lato*), regardless of whether it happens naturally or not. Things get more complicated when CEP is based on nonanthropocentric values for which the level of human intervention may be decisive. For instance, those who defend the preservation of nature are likely to be more reluctant to integrate prescriptive evolution in the CEP toolkit.

## 6 CONCLUSION: THE POTENTIAL OF EVOLUTIONARY POTENTIAL

It would be naïve to believe that all efforts to conserve evolutionary potential at any biological level, no matter how small or big they are, will altogether contribute in harmony to the same grand goal of the conservation of biodiversity. Evolution just does not work that way; equilibriums in biological systems are dynamics and involve trade‐offs and turnovers at all levels, from genes to ecosystems. The truth is, we have tough choices to make and, each time we refer to CEP in a specific context, we should wonder what conservation values it will ultimately serve (or disserve). A tendency of researchers to focus on their own study species, while understandable, could exacerbate the problem. A lesson from the warbler example is that favouring a particular EP vehicle may be at the expense of another.

We suggest that those involved in evolutionary conservation explicitly locate their approach in each of the four dimensions of CEP presented here. To put things clearly though, we are not proposing that every researcher should do this exercise independently, based on her/his personal values. While individual visions and priorities can vary, some ideas appear to be largely shared among individuals, communities and nations, such as reducing the unprecedented rates of species extinction and population declines. Many will also agree that some forms of evolutionary potential are less welcome, such as when organisms have the potential to evolve traits contributing to invasion or making them new vectors of human diseases (Moles, Gruber, & Bonser, 2009). In any case, those involved in conservation should demonstrate why specific proposals to conserve evolutionary potential, in whatever study system, fit with conservation values that enjoy wide acceptance from society, otherwise advocate for the values they endorse. This will imply thinking about vehicles, temporality, measurability and naturalness. It won't be always possible to reach final conclusions about these dimensions. For instance, it may be hard to define the appropriate vehicles or temporal scope to focus on. Yet, we believe that recognizing the uncertainty about these dimensions will make more credible and robust the consideration of evolutionary processes for the conservation of biodiversity.

While our message is actually intended to all those involved in the conservation of biodiversity—scientists, practitioners, policymakers and others—we address it particularly to academic researchers. If science has been a pillar supporting conservation actions, conservation practitioners must also deal with various (economical, political, social) constraints and interests. Academics have more freedom to determine their own research programme and bear responsibility not to lose sight that conservation‐related research must lead to knowledge that is truly helpful to achieve conservation goals, not mere intellectual satisfaction over complex results with extremely hypothetical fallout for conservation. Even rather simple scientific concepts that have been used with some success in conservation, such as management units, are sometimes hard to translate into priorities and prescriptions; they can even be used against conservation, such as when the absence of genetic structure in a species is used to justify policies against the preservation of some populations. Therefore, a concept as complex as the *conservation of evolutionary potential* is even more likely to mislead conservation efforts if its dimensions are not clearly exposed and understood. In good practice, thinking about these dimensions upstream the elaboration of a conservation plan should trigger a legitimate reflection process among stakeholders in any conservation context, helping to identify the right choices for the right context.

**FIGURE 2:**
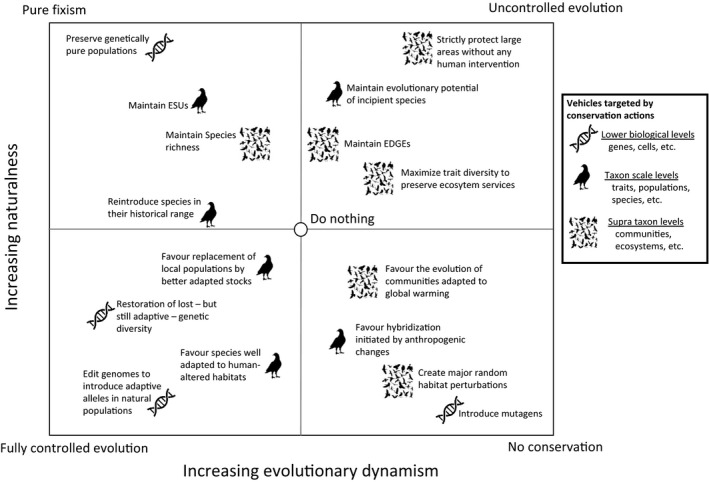
The positioning of conservation actions as a function of the degree of evolutionary dynamism (*x*‐axis) and naturalness (*y*‐axis) they imply. The putative biological level at which lie the vehicles of evolutionary potential is illustrated by icons. The “do nothing” option would be to let human populations continue their development without helping (or harming) biodiversity on purpose. It is used here as the central reference point to position other elements, which are given as examples. The four corners of the box correspond to theoretical extremes. Pure fixism would be to attempt preserving everything *as is* at all levels of biological organizations, with as few traces of human influence as possible. Fully human‐controlled conservation is a situation in which the preservation of biological entities would be insured by constant human interventions on these entities. Uncontrolled evolution is a posture by which the focus of conservationists would be put strictly on future evolution and diversification, without regard to evolutionary history/heritage, nevertheless with the expectation that it will help maintain a certain level of biodiversity. Finally, the “no conservation” extreme falls outside the realm of the conservation of biodiversity as it corresponds to a posture by which both past and future evolutionary histories are not considered at all. It is important to note that opinions on the location of each element on the graph may vary and we invite the reader to try the exercise for herself/himself

Finally, not all imaginable forms of CEP are represented in the discourse on conservation. For instance, as aforementioned one way to conserve evolutionary potential would be to do nothing and let nature evolve freely. Another approach would be to increase EP by generating random mutations, for example through irradiation. We doubt, however, that anyone (including us) would praise for such approaches, because they fall outside the realm of the conservation of existing biodiversity. Nevertheless, along with the genetic essentialism evoked earlier, they set three reference points on a fixist‐evolutionist axis where conservation actions can be positioned (Figure 2). Likewise, actions can be ordered according to their contribution to maintain naturalness. We hypothesize that the more two actions are distant from each other in this space, the more they are likely to be incompatible. Hopefully, this schematization may spark a wider reflection about the implications of the conservation of evolutionary potential.

## CONFLICT OF INTEREST

None declared.

## Data Availability

No data are associated with this paper.
